# Mr. Dyce Sombre on the English Law of Lunacy

**Published:** 1850-07-01

**Authors:** D. O. Dyce Sombre

**Affiliations:** Paris, rue des Pyramides, 2


### MR. DYCE SOMBRE ON THE ENGLISH LAW OF LUNACY.

[WE have received a copy of a work consisting of 589 pages, printed in Paris, and entitled, "*Mr. Dyce Sombre's Refutation of the Charges of Lunacy brought against him in the Court of Chancery.*" It is impossible in the present number (the greater portion of the journal being in type) to give our readers even an idea of the contents of this volume. We shall therefore, on this occasion, merely transfer to our pages, without comment, Mr. Dyce Sombre's "Views of the English Law of Lunacy," with which he concludes his "Refutation."]

"My own painful example must convince every impartial mind, that personal liberty, so jealously guarded in England by the law, is liable to be infringed with the greatest ease on the mere plea of lunacy. By the English law, the Lord High Chancellor, upon petition supported by affidavits, grants a commission to inquire into the state of mind of the party. But can this seriously be considered a sufficient guarantee against erroneous opinions, misrepresentations, or conspiracies? In my case, for example, a physician, as already stated, broke into my apartment, and put me in charge of three keepers, without my having the power either to claim to be heard, or to have recourse to the intervention of a magistrate. More than sixteen weeks was I retained in captivity, before a commission sat in my case; I have already related *how* it sat. The mere word of a physician, supported no doubt by petitions and affidavits, was sufficient to restrain me from the exercise of my liberty—me, who had committed no crime nor illegality, without so much as a question being addressed to me by any responsible person in office, while a thief, a pickpocket or a burglar, cannot be kept twenty-four hours in custody, even when caught in the act, without being examined by a magistrate !

While, in France, the greatest formalities are observed in laying restraint on a man's liberty on the charge of lunacy, an act which cannot be effected without an examination by the tribunal in the presence of the Procureur du Roi (*Code Civil, art.* 496) in England, it is not even thought necessary to ascertain *prima facie* the patient's state of mind by a public officer previously to his being placed under control !

Let us suppose, for instance, that a wife, tired of her husband's presence, and desirous of enjoying her liberty with the command of an easy income, hits upon the plan of making out a case of lunacy against him. She knows all his peculiarities and weaknesses ; she possesses the art of vexing him, putting him into a passion, and inducing him to commit himself by some unguarded threat, which she takes care shall be heard by the menials of the family. This will give her an opportunity of instilling into their credulous minds the discovery she has made, that her husband, poor dear creature, is mad ! He never was so cruel, so violent before ; oh, no ! he is mad.

The stroke takes effect. From this moment all the doomed man's actions are spied and commented upon ; his most innocent and plainest words distorted from their real meaning. An officious doctor is drawn into the secret, he watches like the rest, he registers the fables that are whispered to him about his patient's acts and words ; a well-authenticated threat can be sworn to ; perhaps the lady's promises prompt his zeal. The victim's temper is carefully soured and maliciously excited, his knowledge of family or other affairs cunningly deceived by falsehoods of every shape, and each deception thus imbibed is destined to count fearfully against him on the day of judgment.

At last the day arrives. The petition and affidavits have had their effect ; the physician enters with three keepers, and the devoted man is henceforward a lunatic. He is taken to an asylum, in which the chief doctor, and a brother practitioner who has obsequiously signed the certificate, *have no interest,* as the law directs. From that moment he is as good as in his grave ; his papers are taken from him, his money and property removed, his room is inaccessible to the visits of a friend, save the benign household god of the place ; who is delighted at the capital prize his friend Dr. N. has procured him. Meanwhile, care is taken to provide further evidence of the patient's state of mind. Loss of liberty and anxiety are excellent prescriptions to make a man mad, if he is not ; but they may not be enough. New annoyances must therefore be invented ; his sleep must be often disturbed by the keepers ; if he complain, it is clear that he believes in ghosts. His food may be so regulated as to produce dyspepsia, a complaint likely enough to arise from mere confinement, and no less likely to produce unfavourable effects on the brain. Paper and writing materials are freely left in the power of the victim; for it is ten to one that ennui and seclusion will dictate to him some nonsense which may be brought in evidence against him. At last, however, after sixteen weeks' schooling in the art of running mad, the commission-day draws near. Then comes the triumph of the mad-doctor; for if he be not an arrant dunce, and if he understand to turn an honest penny, he will just mix the slightest possible dose of henbane or any other stupefying substance with the food of the pretended lunatic during the last few days, a process which will greatly contribute to make him unfit to defend his case before the commission.

Meantime the lady will have taken the utmost pains to lament her hard fate in every body's presence. The news flies from mouth to mouth: Mr. Such-a-one is mad! and by the time the case is to come on, there is little necessity of using much ingenuity in packing a jury, the public mind being sufficiently prejudiced already to render that precaution unnecessary.

The victim is called in; the mass of false or adulterated evidence collected in all this time is laid before the commission, who see all through the magnifying-glass of pre-conceived prejudices; and should the patient attempt to defend himself, the foreman of the commission perhaps will answer: "Oh, we want to hear no more, we have heard enough!"

Though in drawing this picture, I have made allusion here and there to circumstances that actually existed in my case, I may confidently state that such proceedings are not unfrequent; nay, I might quote an instance of an English lady of high descent, who, in 1843, attempted to rid herself in the manner here described, of her husband, in a provincial town of Italy; which attempt was fortunately averted by the bold interference of a gentleman who was little better than a stranger to the family. This very distant allusion would suffice to recal the fact to the memory of the parties in question.

The sentence of lunacy once pronounced, the doomed man becomes the doctor's slave. He has no hopes of being freed from his meshes. All he says or does, is dictated by lunacy; if a reasonable act is admitted, it is attributed to a lucid interval; in short, unless the patient succeed in making his escape, he runs a very good chance of losing his reason in good earnest, to the no small triumph of the honest practitioner.

But if he effects his escape, he will follow my example, and apply his first moments of liberty to prove by the testimony of other physicians how wronged he has been. But what are the most eminent physicians of Paris, Petersburgh or Brussels, to Sir J. Clark, Drs. Southey and Bright? Mere school-boys, of course; for have they not dared to question upon scientific grounds as well as matter of fact, the validity of the sentence of those three great men, whose opinion has been so logically set forth in the numerous reports already given, and which, for the sake of the reader's complete edification, I will just examine a little more nearly.

It is a singular fact, that whereas generally the hallucinations of a lunatic are clearly defined, my doctors have never been able to give a well-defined name to mine. I am accused of excess of jealousy, of suspicions against innocent persons, of violence, want of memory, and what not. But even supposing all these accusations to be true, can they possibly constitute lunacy? A lunatic will think he sees, or hears, or knows, or is, something that cannot be seen, or heard, or known, or be, on account of some *physical* impossibility; but if suspicions of things *physically possible* are classed as hallucinations, where, in the name of Heaven, can you draw a line between *sanity* and *insanity?* Again, if I were accused of admitting *incompatible* things as *compatible*, or declaring the contrary, I might be guilty; but are the imputations I formerly brought against Mrs. D. S. of that nature? If I was mistaken in the facts, was that a reason for declaring me mad? were those imputations *impossible?* Nay, more, could the doctors, who are so positive in pronouncing my lunacy, *prove* by substantial facts, that such imputations were really *false?*— They might *judge* them false; but as to proofs, though I might be at a loss to prove my allegations, I suspect they would be equally puzzled to prove the contrary.

If jealousy be madness, if those who doubt a woman's virtue are to be considered lunatics, then either England is the chastest country in the world, or else I would recommend that the whole face of the country be covered with madhouses

"Thick as the leaves of Vallombrosa."

Drs. Southey and Bright, in their report of July 24, 1844, gravely enumerate all the heinous offences I am accused of. The first and principal one I have just disposed of. Next comes my threat that I would cut off Mrs. Dyce Sombre's nose. The charitable doctors do not attempt to suppose that such a threat might possibly have escaped me in a moment of passion, caused by the superabundant and probably sharp eloquence of my partner; they seem to forget that such threats generally fall thick as hail-stones in matrimonial squabbles, without the remotest intention on either side of ever putting them into execution. Oh no, that does not strike them; pronouncing me a lunatic is by far the easier course of the two.

My determination never to be re-united to my wife, though on a former occasion I had proposed certain conditions on the performance of which I would consent to receive her again, is considered a proof of lunacy. None but a lunatic, according to Drs. Southey and Bright, can change his mind. For then the proverb "*Second thoughts are best*" is lunacy. My not thinking it a hasty step to send a challenge to Sir F. B. upon mere suspicion, is lunacy. Were Drs. Southey and Bright to read the papers, they would find, however, that almost all duels fought on account of ladies are owing to mere suspicions.

Though not mentioned in the present report, I may here advert to the strange accusation Sir J. Clark brought against me before the commission of lunacy, namely—that I believed in ghosts. I have already flatly denied this idle imputation. But what if it were true? Would that prove my insanity? How many devout persons there are—not to mention the peasantry of almost every country in the world,—how many well-educated persons believe in visible spirits, in visions of saints, in second sight, somnambulism, prophecies and such like, while nobody in their senses would dream of calling them mad? The ghosts *I* did see, were of flesh and blood, none else but the keepers, who out of extraordinary regard for my welfare, for which I am profoundly grateful, used to come and startle me from my sleep. So much for the veracity of the "reports."

Next comes the imputation that I imagined my food had been drugged. Lunacy again! incorrigible lunacy! When a man who generally feels himself well, one day feels, on rising from dinner, certain unusual symptoms, unlike what he ever felt before, is it surprising that he should believe that something he has eaten has disagreed with him? And when he finds himself unjustly under restraint on a pretext of lunacy, surrounded by persons whose behaviour to him has been anything but prepossessing, is it strange that he should suspect them of foul play? My surmises may have been wrong; I may have been mistaken; but is a mistake a hallucination? is it lunacy? Let the impartial public answer.

I now come to the report of September 26th, 1846. The doctors are happy to find that my food was *not* drugged at Brussels, but to their utter mortification they find that I will *not* live again with my wife, that I still believe in her misconduct, and even name the persons on whom my suspicions rest, and my reasons for them. Now I humbly submit that the best course to cure me of my inveterate lunacy on this ticklish subject, would have been to convince me by some substantial proof or other, that my suspicions were not borne out by appearances. The persons I accused might not have been in the country at the time, or they might have avoided the society of my wife, or my informants might have been maliciously intent upon deceiving me? Nothing of the kind, however, the sapient doctors attempted to prove or even to inquire about; they did not fail however to notice "my peculiar look and manner" with which I answered *no* to their question concerning the illicit intercourse between Lord St. Vincent and his daughter, and to decide that "my words were at some variance with my thoughts." What penetration to be sure!—But on the following day they are surprised to hear that I admit "that my notions of Mrs. Dyce Sombre's infidelity were all delusions" and that "she is as virtuous and chaste a woman as ever lived." With the same wonderful penetration they are so conspicuous for, they find that I "seemed glad to have disburthened myself of a disagreeable task." I am rather surprised they did not in this instance express a suspicion that I had been in the interval advised by somebody to take that course, or that I might myself have thought it advisable to cut the matter short at once by doing so. But they remark that I still continue "to talk of Mr.

C. F. and Gen. V. as persons who had deeply injured me." I have already mentioned the facts relating to the latter, and shall not therefore return to the subject. But as to what I said of the former, what does that prove, except that I am a bad adept in dissimulation, and that the suspicions I then entertained were sufficiently strong to throw me off my guard, when the legitimate jealousy of a husband was distorted by evil-intentioned persons into a proof of lunacy? Did the French physicians, did the Prefect of Paris consider jealousy, even though unfounded, a sufficient reason for locking up a person in a lunatic asylum, or even depriving him of the control of his property?

The doctors, indeed, attribute my contradictory answers to an attempt to "suppress the latent delusion;" but the question is: Can it be called a delusion at all? Can an unfounded suspicion be called a delusion in the sense in which it is applied, to establish lunacy? If it can, then the number of lunatics must comprise the immense majority of the nation!

But luckily for me, the doctors admit that I no longer believe in ghosts, nor that my food is mixed with poison!

They conclude with a modest insinuation, that their opinion is at variance with the "testimony of various medical men in this country and also on the continent," owing to their being ill-furnished with the facts and early history of the case; or in plain English, that Drs. Southey and Bright are the only persons in the world able to judge of the state of mind of Mr. Dyce Sombre. There is but one God, and Drs. Southey and Bright are his prophets!

I shall refer the reader to the French report, where it will be seen that Sir J. Clark had kindly volunteered every possible information on the subject, including the East India Company, the Archbishop of Canterbury, and all the vile trash concocted against me, not omitting the very conclusive notes written in my own hand. The physicians of St. Petersburgh had the benefit of the same facts from the French report.

The report of August 5th, 1847, begins with a rich specimen of logic. The doctors set out with a statement that the difficulty of making a satisfactory report of my case is "not diminished." Still, in the next sentence, they admit that I have "acquired much more self-control." In that case I humbly submit, there must have been *some* diminution in the alleged difficulty.

But the reason why the difficulty still subsists, is probably that "to some of the allegations contained in the affidavits I gave a positive denial, others I endeavoured to explain away."

A great proof of lunacy, certainly! here are affidavits regularly signed and sworn, to which I give a flat denial! Of course, being a lunatic, I *must* be wrong; the doctors do not hesitate about whom they have to believe; false, mistaken, or ungrounded affidavits are quite an impossibility!

But "others I endeavoured to explain away!" Now, is not that a proof of that peculiar cunning so frequently met with in such confirmed lunatics as I am? But I did not succeed in my attempt; no, the doctors tell you, I only "endeavoured." Whether the grounds upon which I "endeavoured" were good or bad, the doctors took no trouble to ascertain; for am I not a lunatic?

I admitted, however, "substantially the whole of the statement made by Signor Solaroli." The doctors do not think it worth while to remark that that was a proof of memory. I further "asserted positively" the illegitimacy of Madame Solaroli, quoting Lord Metcalfe's and Mr. Prinsep's authorities. Now mark the doctors:

"We regret that we have no means of ascertaining this question of fact, as, IF UNFOUNDED, it would throw much light upon the 'state of Mr. Dyce Sombre's mind.'"

How candid! IF UNFOUNDED, I should still be a lunatic, of course; —ah, but IF FOUNDED? The doctors do not venture to contemplate such a possibility as a lunatic being right in spite of the Faculty!

The reader has already seen, that if I had not positive direct proofs on the subject of Madame Solaroli's illegitimacy, I have pretty substantially established that the matter was generally suspected, independently of my personal knowledge of the matter, which is considered of no value, because "I am of unsound mind."

The doctors then proceed to state that "such groundless aspersions (upon Messrs. Vizard, Leman, etc.) on the characters of gentlemen of unquestionable integrity and honour, appear to us to be marks of the same mental disorder which led him to suspect the fidelity of his wife."

We have the doctors' word for it, that "such aspersions" are groundless; of course they have minutely examined the persons in question, or witnesses to the subject; else how could they know that they are groundless? Have not we seen men of immense respectability hanged for forgery, nay, murder? a bishop forced to fly from Ireland under the conviction of an unnatural crime? members of parliament convicted of bribery? Do we not daily see in the papers men of rank or property in every station of life called before the justice of the country to purge themselves of accusations of seduction, rape, embezzlement, breach of trust, crim. con., perjury, calumny, and every item almost of the catalogue of crimes and misdemeanours, —and do we not almost always see verdicts of "Guilty" recorded against them? And is it not generally felt and known that the number of treacherous, dishonourable, and shameful acts which the arm of the law is by far too short to reach or to punish, is infinitely greater than those offences which come to light?—And with such facts staring you in the face, you, Drs. Southey and Bright, find it in your consciences to declare a man a lunatic, because, betrayed, cheated, swindled and persecuted as he has been for the last twelve years, as proved by facts and documents, he has learnt to suspect the motives and actions of those with whom he has to do, till he has some direct proof of their honesty? And if, as you pretend, suspicion be a mental malady, who, I ask, has fomented mine more than you, who have tasked your ingenuity to the utmost, in order to represent my simplest words or actions as symptoms of a diseased intellect?

The doctors then proceed to state, that having made a written declaration to exonerate my wife from all suspicion, I had confirmed it in their presence, but refused to live again with my wife, on account of her temper. Upon which my wise examiners give birth to the two following sentences, which I beg to put face to face:

"It is difficult to believe in the entire removal of the delusion in question whilst any feeling hostile to Mrs. Dyce Sombre seems to subsist, etc."

"It is very satisfactory to us that Mr. Dyce Sombre has admitted that he laboured under delusions up to the time when we parted with him at Dover, thus proving the correctness of the opinion we formed, etc."

Now if, as the doctors insinuate, my admission that I was wrong in my surmises concerning my wife's infidelity, was insincere, if it was an effort to deceive them, what on earth can that prove as to the correctness of their opinion? How can a falsehood be adduced in evidence of a truth? But if, on the contrary, my admission was sincere, and corroborates their former opinion, it is unwise in them to invalidate such testimony by throwing a doubt on its sincerity; while at the same time it is ungenerous to deprive me of the benefit of it, as a proof that the capital charge against me, stupid as it is, is removed.

Let me now compare the three following passages:—

REPORT

*Of September 26th*, 1846.

"We are still convinced, that his mind continues to be impressed with the notion of his wife's infidelity, &c."

REPORT
*Of August 5th*, 1847.

"If on our last examination of this gentleman we found it difficult to make a satisfactory report, on the present occasion our difficulties are not diminished."

"In conclusion we are bound to admit, that we were unable to elicit any positive delusion under which Mr. Dyce Sombre labours."

After perusing the first passage, the reader must think that the delusion complained of in 1846, has been found equally strong in 1847, since the doctors' "difficulties are not diminished." Still the third passage denies the latter assertion; since it contains a reluctant avowal that they "were unable to elicit any positive delusion." Now which of the two declarations are we to believe? One thing is certain in my opinion; that is, that neither Locke nor Harris presided over the framing of the doctors' reports.

But the scrupulous practitioners add immediately after, that they "feel no confidence that he is entirely free from such delusions." Therefore, because Drs. Southey and Bright, although confessedly upon no positive grounds whatever, "feel no confidence," a man is to be kept from his property, an outlaw from his kingdom, debarred from the redress the meanest subject of the British empire has a right to demand! What would the public say if a jury in a case of burglary or murder, were to frame a verdict as follows:—"We cannot find the prisoner guilty, although we have no confidence in his innocence?" Him the judge would instantly acquit; but I, a presumed lunatic, am still an exile, with the prospect of a prison, if ever I set foot without permission within the jurisdiction of the Lord High Chancellor, and merely because two doctors, in the teeth of evidence, declare they have no proofs, but still can have "no confidence."

The next passage is remarkable :—

"When we consider the length of time during which his malady has continued, and the self-command by which he has been enabled to deceive so many physicians both foreign and English, we cannot but hesitate in giving credence to his own statements; nor, because he tells us that in September last he became satisfied of the injustice of his notions respecting his wife, can we therefore conclude that he is now perfectly sane."

This is rich : I will but call it self-conceit—the dictionary might furnish me with a much more appropriate term. Here are two men calling themselves sane, who, after having but a moment before confessed they had no proofs, sweepingly declare in the face of the world that the most eminent physicians of the continent specially entrusted with the cure of mental disorders, and many eminent men of their own country, were all duped. And by whom? by a lunatic, whom they alone, H. H. Southey and J. Bright, are competent to fathom! Waiving all considerations of the futility of the symptoms on which my lunacy is supposed to rest, surely my madness, if capable of deceiving so many learned men of four countries, must be infinitely preferable to the sanity of my doctors!

I now come to the Report of November 18th, 1848, signed by Drs. Southey, Bright, Clark, and Martin.

Here a mistake, concerning the date of my change of opinion respecting my wife's virtue, is maliciously pointed out as a mark of insanity, and my sincerity is again questioned. Next follows this remarkable passage :—

"He mistakes the creations of his own fancy for facts, and reasons upon them accordingly."

Here I will just ask the following question :—

Is a man who labours under a mistake a lunatic? If so, I venture to say there is not a man in the world who is not, or has not been, a lunatic.

If not, then pray what does a man do when he labours under a mistake? Does not he take it for a fact, "and reason upon it accordingly?"

Nay, what have my doctors done? Have they not mistaken the "creation of *their own* fancy," my lunacy namely, for "a fact," and have they not "reasoned upon it accordingly?"

As to whether what they call the "creations of my own fancy" are facts or not, the doctors have not taken the trouble to inquire. I have given sufficient explanations in former parts of this Refutation respecting the circumstances they adduce as proofs of my insanity; but the manner in which they expose the affair of Lord Ward, notwithstanding the very clear explanation I gave them of the matter, and which I have already given here, is a clear act of downright malevolence. The matter is maliciously related, my explanation completely suppressed, and a hint cleverly thrown in, that perhaps "the whole transaction is a matter of imagination!"

The same may be said of the manner in which they give an account of our conversation respecting Madame Solaroli's illegitimacy. I have given so much evidence on the subject in this Refutation, in the shape of argument, letters, and affidavits, that I need only revert to the impudence with which my assertion respecting Mr. Glynn is declared to be a delusion.

The rest of the report is of the same character as the preceding ones, with this difference, that whereas the latter seem rather dictated by conceited self-importance, spite and dishonesty appear to have had a great share in this.

When we compare the dignified, precise, and scientific character of the reports of Paris, St. Petersburgh, and Brussels, with the shuffling, uncertain, prejudiced, and malicious tone of the reports of these English physicians, we cannot help entertaining the idea, erroneous as it may be, that the latter have been prompted by some secret motive, which however in the present state is not worth inquiry.

The reports I have here examined may be condensed as follows:

I believe in the unchastity of my wife; therefore I am a lunatic.

I believe in the illegitimacy of Madame Solaroli; therefore I am a lunatic.

I was, from ignorance of the worldly station of Lord Ward, led into a mistake; therefore I am more a lunatic than ever.

Thus it is, that by the combined efforts of intrigue, ignorance, and misrepresentation, and by the defective state of the English law as regards lunatics, I am debarred from personal liberty in my mother-country; the management of my property is withheld from me, while it is wasted through negligence or cupidity, and myself cast out, as far as practicable, from the society of reasonable men, a lunatic among the sane, by the mere dictum of a few men, who openly profess to set their own wisdom against that of the rest of the world.

And all this in a country which prides itself upon being the only one in the world where personal liberty is fairly understood—where a pickpocket or a murderer will meet with all the tenderness of the law; but where, alas! there is no law for a presumed lunatic, when there are interested parties, whose wishes are that he should remain so.

With these I conclude, and leave the public to form their own opinions on my case; and whether such persons as the Chancery Doctors have proved themselves to be, are worthy of the trust the Lord Chancellor reposes in them, and whether the Lord Chancellor ought not at once to annul, and crush the Commission of Lunacy under which I now labour.

D. O. DYCE SOMBRE.

